# Effect of Different Doses and Times of FK506 on Different Areas of the Hippocampus in the Rat Model of Transient Global Cerebral Ischemia-Reperfusion

**DOI:** 10.1155/2019/8047672

**Published:** 2019-07-29

**Authors:** Zahra Nadia Sharifi, Hamid Zaferani Arani, Maedeh Olya, Hesam Adin Atashi, Shabnam Movassaghi

**Affiliations:** ^1^Department of Anatomical Sciences and Cognitive Neuroscience, Faculty of Medicine, Tehran Medical Sciences, Islamic Azad University, Tehran, Iran; ^2^Cognitive and Neuroscience Research Center (CNRC), Amir-Almomenin Hospital, Tehran Medical Sciences, Islamic Azad University, Tehran, Iran

## Abstract

**Background:**

Stroke is a major worldwide problem that is leading to a high mortality rate in humans. Ischemia, as the most common type of stroke, is characterized by tissue damage that can occur due to insufficient blood flow to the brain even for a brief duration, leading to the release of inflammatory factors, cytokines, and free radicals. In this study, we investigated the effective dose and injection time of FK506 as an immunophilin ligand for providing a suitable effect on cells of CA2, CA3, and dentate gyrus of the hippocampus.

**Methods:**

In this in vivo study, a total of 48 male Wistar rats were divided into nine groups. The ischemia model was induced by the ligation of bilateral common carotid arteries. The doses of FK506 (3, 6, and 10 mg/kg) were administered intravenously (IV) at the beginning of reperfusion, followed by repeated injections (10 mg/kg) at 6, 24, 48, and 72 hours after ischemia, respectively. Brains were removed and prepared for Nissl staining and the TdT-mediated dUTP Nick End Labeling method.

**Results:**

Data showed that global ischemia did not decrease the number of viable pyramidal cells in CA2 and CA3 regions, but significant differences were observed in the number of viable granular cells and apoptotic bodies in the dentate gyrus between the control and ischemia groups. Repeated doses of 6 mg/kg of FK506 at an interval of 48 hours were deemed to be the suitable dose and best time of injection.

**Conclusions:**

It seems that FK506 can ameliorate the extent of apoptosis and may be a good candidate for the treatment of ischemia-induced brain damage.

## 1. Introduction

Hippocampus is a small part of the human brain which plays roles in memory forming, organizing, and storing. It is part of the limbic system structure and anatomically has four main parts called dentate gyrus, Cornus Ammonis (CA) 1, CA 2, and CA 3 [1].

Stroke is a major contributing factor for brain ischemia and the third leading cause of mortality in Western countries. Brain ischemia leads to movement, visual, sensory, and behavioral disorders, especially aphasia and impaired spatial learning [2–4].

Reperfusion injuries refer to the tissue damage caused by the restoration of blood to a tissue after a period of ischemia; a special condition is caused by the absence of oxygen and nutrients during the period of ischemia, in which inflammation and oxidative damage result from the induction of oxidative stress once the blood supply is restored [5–7].

Reperfusion plays a major role in different stages of brain ischemia, which is observed in various types of strokes and traumatic brain injuries [8].

Excessive inflammatory response, apoptosis, activation of catalytic enzymes, release of other oxygen-free radicals, and changes in gene expression patterns are the most significant pathological events occurring during ischemia [9–11].

Studies on experimental models have proven the protective effect of more than 100 materials on the programmed cell death [12]; however, despite the promising results of experiments on animal models in preventing this type of cell death, unfortunately, no effective medical therapy has yet been found to deal with brain ischemia. This may be due to the lack of efficacy or side effects of drugs [13].

It has been found that some of the materials mentioned above have protective effects against neurotoxicity, and a subsequent increase in glutamate, and accordingly in immunophilins as the agents protecting neurons against damage, has received much attention [14].

Immunophilin ligands are compounds that are useful in the treatment of neurological injuries and diseases. They cross the blood-brain barrier and are orally bioavailable. As a result, the use of immunophilin ligands has recently been considered as a suitable new strategy for protecting the nervous system. In humans, as immunosuppressive drugs, two ligands, namely, cyclosporine A and tacrolimus (known as FK506), are used. The protective effects of both ligands have been proven; however, only FK506 has neurogenic activity [14–16].

FK506 is an agent derived from bacteria* Streptomyces tsukubaensis *that is used as an immunosuppressing drug [17]. Many functional roles of FK506 have been reported; some of them are the calcineurin-dependent and calcineurin-independent mechanisms. According to previous studies, the protective effect of FK506 is associated with calcineurin-dependent mechanism, which results in the reduced production of nitric oxide; also, its neurogenic activity is associated with its calcineurin-independent functions [18].

Despite a large number of reports on the protective effects of FK506 on different tissues [19, 20], few studies have investigated the neurotrophic properties of this drug on different parts of the hippocampus. Hence, in this study, we investigated the neurotrophic effect of different doses and different times of FK506 injection on the CA2, CA3, and dentate gyrus regions, subsequent to global ischemia/reperfusion (I/R) in the male Wistar rat.

## 2. Materials and Methods

### 2.1. Animals

This in vivo study was conducted on 48 male Wistar rats, weighing 250 g to 300 g. Animals were obtained from the Pasteur Institute, Tehran, Iran, and kept in standard cages under 12 hours of lighting and 12 hours of darkness, 22°C to 24°C temperature, and a humidity of 45% ± 5 %. Sufficient amounts of food and water were made available to the animals.

### 2.2. Surgical Procedure

Animals were anesthetized using intravenously (IV) injected sodium pentobarbital (40 mg/kg). A vertical incision was made in the anterior region of the neck. Sternocleidomastoid (costoclavicular) muscles were pushed away to expose the common carotid arteries on both sides. After separating the vagus nerve, the arteries were closed by the microsurgery clamp of Yasargil for 20 min followed by reperfusion. During the surgery, rectal temperature was regularly measured via a thermometer and was stabilized at 37°C ± 0.5°C using a heat lamp.

### 2.3. Groups and Design

Rats were randomly allocated into nine equal groups (n=6 per group) as follows:

Control group: rats underwent anesthesia by 40 mg/kg (IV) sodium pentobarbital. In the I/R group, animals underwent the I/R model. Experimental (E) group 1 received the 10 mg/kg dose of FK506 (IV) at the beginning of the reperfusion phase. In E2-E5 groups rats received the 10 mg/kg dose of FK506 (IV) at the beginning of the reperfusion phase and a repeated dose at 6, 24, 48, and 72 hours after ischemia, respectively.

Our previous study [21] showed that the injection of FK506 at the 48-hour interval was the most appropriate time of injection for recovering of the neurons region after ischemia. Hence, in the E6 and E7 groups, 3 and 6mg/kg doses of FK506 were used to determine the best dose of injection. All the doses were selected based previous studied [22, 23].

### 2.4. Histopathological Studies

Animals were euthanized 96 hours after ischemia and, then, transcardially perfused with PBS followed by perfusion with 4% paraformaldehyde at pH 7.4, and the brains were postfixed at 4°C for three days, embedded in paraffin [21].

Paraffin-embedded coronal sections at different thicknesses were cut for different staining methods, 10 and 3* μ*m thickness for Nissl staining and TdT-mediated dUTP Nick End Labeling (TUNEL) staining, respectively, between 2.3 and 5mm posterior to bregma fortune.

#### 2.4.1. Nissl Staining

After fixation and preparation, coronal sections with a thickness of 10 *μ*m and a distance of 2.3 to 5 mm from the posterior of bregma were prepared using a rotary microtome placed on the gelatinized slides and stained using the Nissl method. The samples were analyzed using an optical microscope with 400× magnification. Only neurons with clear nucleus and nucleolus were considered as viable and healthy cells. From each sample, eight photomicrographs with a minimum distance of 40 *μ*m were randomly selected. Pyramidal cells in the CA2 and CA3 regions and granular cells in the dentate gyrus area were counted by Image Tools 2 software, and the mean counts were determined [21, 24].

#### 2.4.2. TUNEL Staining

After fixation and preparation, coronal sections with a thickness of 3 *μ*m and a distance of 2.3 to 5 mm from the posterior of bregma were prepared using a rotary microtome and placed on Silane slides. To characterize the apoptotic cells, we performed TUNEL staining using TUNEL kits (Roche, Mannheim, Germany) according to kit's instructors. Briefly, sections were deparaffinized in xylene, hydrated by decreasing gradients of ethanol gradient, then washed in phosphate-buffered saline (PBS), and permeabilized by proteinase K (20 *μ*g/ml) for 30 minutes at room temperature. Sections were incubated with 3% H2O2 in methanol for 10 minutes to block endogenous peroxidase (POD). Then each section was incubated in the TUNEL reaction mixture at 37°C for 60 min, rinsed with PBS, and visualized by using Converter-POD for 30 min at 37°C with a humidified atmosphere. Sections were rinsed with PBS, and then 50-100*μ*l diaminobenzidine (DAB) substrate was added and rinsed with PBS. From each sample, four photomicrographs with 400× magnification were provided, and three photomicrographs were randomly selected. The cells in the CA2, CA3, and dentate gyrus regions were counted with the help of Image Tools 2 software [21, 24].

### 2.5. Statistical Analysis

Data were analyzed using Statistical Package for the Social Sciences Version 16 (SPSS Inc., Chicago, IL, USA) statistical software via one-way analysis of variance and Tukey's statistical tests. Also, normality was tested using the Kolmogorov-Smirnov test. The significance level was set at P≤0.05.

### 2.6. Ethical Approval

All applicable international, national, and/or institutional guidelines for the care and use of animals were followed.

## 3. Results

**Figure 1:**
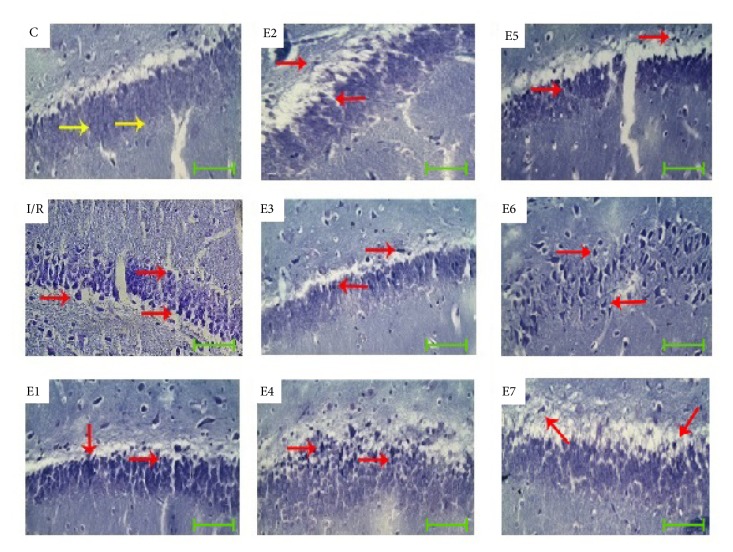
Photomicrographs of coronal sections of the hippocampus (dentate gyrus area) showed that in the control group (C), pyramidal cells (yellow arrows) showed normal morphology. In the I/R group, ischemia induces shrinkage, apoptotic, and fragmented neurons (Nissl bodies, red arrows). However, administered FK506 could significantly reduce Nissl bodies in E1-E5 groups (Nissl staining, 400× magnification, scale bar= 50 *μ*m).

**Figure 2:**
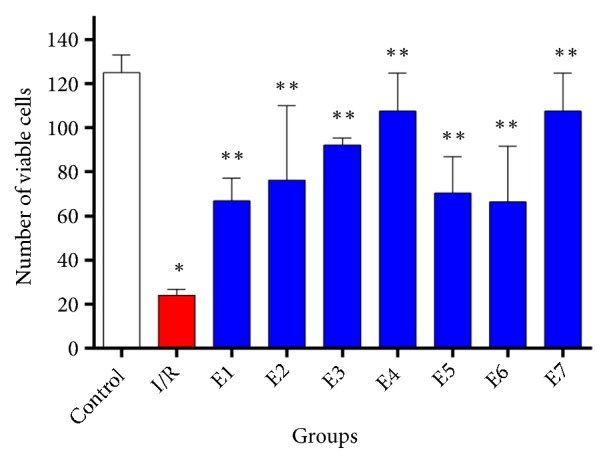
The quantitive analysis of Nissl staining indicated that, in I/R group, the number of viable cells significantly decreased in the comparison control group. Also, received FK506 ameliorated this reduction in all experimental groups. In addition, number of survived neurons in E7 is higher than those of other groups, indicating the best treatment dose. Data are presented as mean±SD. *∗*P≤0.001 vs. control group, *∗∗* P≤0.05 vs. I/R group.

Results showed that clamping the common carotid arteries for 20 min could not reduce the number of viable pyramidal cells in the hippocampal CA3 and CA2 regions, so there was no statistically significant difference between the control and ischemia groups (P=0.659). However, ischemia caused a marked reduction in the number of viable granular cells in the dentate gyrus region, and a significant statistical difference was seen between the control and I/R groups (Figure 1). The quantitive analysis showed that there was a significant decrease in survived neurons between the control and I/R groups (P≤0.001, Figure 2). However, this reduction was markedly improved by administrated FK506 in rats at E2-E7 groups in which this achievement was more significant in the E7 group, which indicated that a repeated dose of 6 mg/kg of FK506 was the best treatment dose.

**Figure 3:**
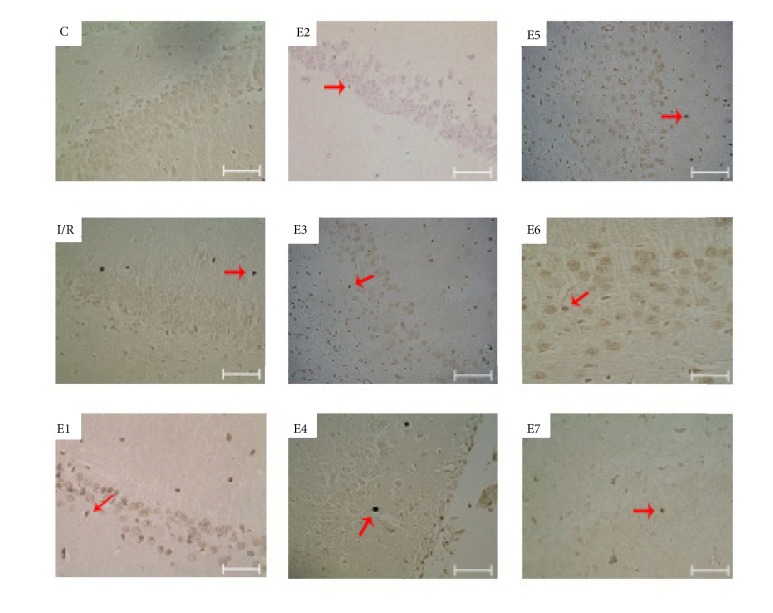
TUNEL staining of rats' hippocampal dentate gyrus area. There were high apoptotic bodies (TUNEL-positive cell) in I/R group in comparison with control group (TUNEL-negative). Also, treated rats with different dose of FK506 at different time showed decreased TUNEL-positive cells (red arrows) in comparison with I/R group. However, this change was not statistically significant in E1, indicating that a repeated dose was needed for the protective effects of FK506 against ischemia (400× magnification, scale bar= 50  *μ*m).

**Figure 4:**
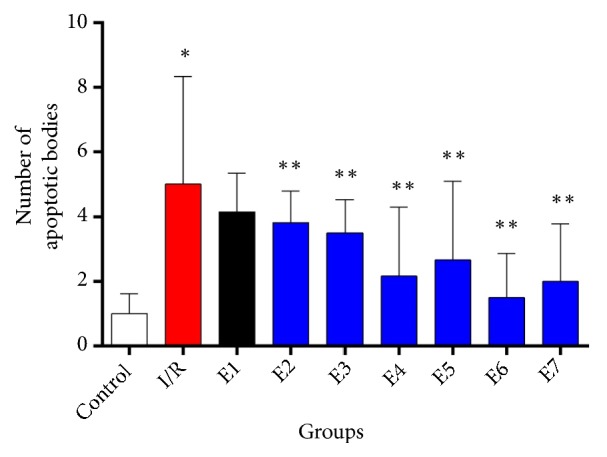
Effect of different doses of FK506 on the number of apoptotic cells in the hippocampal dentate gyrus area. Results revealed a significant difference between control and I/R group. Although there was no significant difference between I/R and E1 group, FK506 treatment could cause considerable reduction of apoptotic bodies in E2-E7 groups. Data are presented as mean±SD. *∗*P≤0.05 vs. control group, *∗∗* P≤0.05 vs. I/R group.

Data from TUNEL staining showed a significant difference between the experimental and control groups, except E1 group, in terms of the number of apoptotic cells in the dentate gyrus area, after I/R induction (P=0.016, Figures 3 and 4). Accordingly, the mean number of apoptotic cells increased after ischemia and decreased after the administration of FK506 (Figure 4). There was no statistically significant difference between the I/R group and E1 group (P=0.26). Indeed, based on the results of data analysis, to decrease apoptotic cells, it is necessary to inject two doses of FK506.

## 4. Discussion

Many studies indicate that acute ischemia and subsequent reperfusion induce considerable cell death in mammals. The cytotoxic lesions show their effects, especially in the hippocampus and motor cortex [24–26].

Neurons in the brain require a lot of oxygen; mature neurons are more vulnerable to ischemia [27]. Granular cells in the dentate gyrus and hippocampal CA1 regions are very sensitive, and they quickly react to global I/R [27, 28].

Immunophilins ligands have recently been considered as a neuroprotective agent. It has been recommended that they should be used for the treatment of neurological lesions and diseases. It can be stated that the most crucial goal of this compound is to inhibit calcineurin [29].

FK506 is one of these ligands. Calcineurin is an essential enzyme that plays a major role in regulating the phosphorylation of many proteins. The concentration of calcineurin in the brain is about 3 to 10 times higher than that in other tissues [30, 31]. FK506 has a high tendency to bind with FKBP12 and make FKBP-FK506 compound, which has the ability to inhibit calcineurin [32]. There are a few reports available about the protective effect of the drug at the tissue level in different regions of the hippocampus. Hence, in this study, we evaluated different areas of the hippocampus at different times of injection with varying doses of the FK506 to determine the best time of injection and to identify their effects on different regions of the hippocampus.

The results of our study showed that the administration of a single dose of the drug (10 mg/kg) exactly at the time of reperfusion did not increase the number of viable granular cells in the dentate gyrus. Besides, this dose did not reduce apoptotic cells in the injured area of the hippocampus. However, when the same dose of the drug was repeated after 48 hours, the number of viable cells was significantly increased. It presents FK506-reduced ischemic effects on the degenerative hippocampal neurons. FK506 can suppress neuronal nitric oxide (nNOS) and after that induced NOS. This procedure may contribute to the neuroprotective effect of FK506 [21].

Our findings showed FK506 as a neuroprotective agent that can reduce the delayed death of neurons. We also found that the repeated injection of the FK506 could lead to a decrease in apoptotic cells in the ischemic region. These findings are consistent with the results of the previous studies that demonstrate the protective effect of FK506 [22, 33].

Some studies demonstrated that FK506 could reduce microglia activities, and this suppression by FK506 may prevent the damage of ischemia. Microglia releases some important cytokines such as reactive oxygen radicals and nitrogen intermediates, as well as some other types such as interleukin-1*β* and tumor necrosis factor-*α* [34–36].

The use of FK506 and its derivatives such as L-685818 can improve severe sciatic nerve damage via recovering of nerve function and regeneration of the myelinated nerve fibers [37]. Our findings similarly showed that the FK505 could increase the number of granular cells in the dentate gyrus after the induction of global transient I/R, which largely depends on the time of injection.

The mechanism that enables FK506 to prevent ischemic brain injuries has not been fully recognized yet; probably, this mechanism is more dependent on new protein synthesis than on calcineurin inhibition [38, 39]. Another hypothesis explains that the suppression of NOS may reduce ischemic damage by the procedure of inhibition of N-methyl-D-aspartate receptors that have neurotoxicity [40].

As the main limitations of our study, we even updated only histopathological studies and could not examine functional and/or behavioral test to show a comprehensive comparison of FK506 on rats. Hence, we recommend that future studies with both histological and behavioral tests to illustrate a better comparison are needed.

## 5. Conclusions

FK506 neurotrophic and protective properties can improve I/R damage with different dose as well as the varying time of injection, and it is used as a treatment option for ischemic brain lesions.

## Data Availability

The data used to support the findings of this study are available from the corresponding author upon request.
